# COVID-19: Integrating the Complexity of Systemic and Pulmonary Immunopathology to Identify Biomarkers for Different Outcomes

**DOI:** 10.3389/fimmu.2020.599736

**Published:** 2021-01-29

**Authors:** Thais Fernanda de Campos Fraga-Silva, Sandra Regina Maruyama, Carlos Arterio Sorgi, Elisa Maria de Sousa Russo, Ana Paula Morais Fernandes, Cristina Ribeiro de Barros Cardoso, Lucia Helena Faccioli, Marcelo Dias-Baruffi, Vânia Luiza Deperon Bonato

**Affiliations:** ^1^ Basic and Applied Immunology Program, Ribeirao Preto Medical School, University of Sao Paulo, Ribeirao Preto, Sao Paulo, Brazil; ^2^ Department of Biochemistry and Immunology, Ribeirao Preto Medical School, University of Sao Paulo, Ribeirao Preto, Sao Paulo, Brazil; ^3^ Department of Genetics and Evolution, Federal University of Sao Carlos, Sao Carlos, Brazil; ^4^ Department of Chemistry, Faculty of Philosophy, Sciences and Letters of Ribeirao Preto, University of Sao Paulo, Ribeirao Preto, Brazil; ^5^ Department of Clinical Analysis, Toxicological and Food Science Analysis, School of Pharmaceutical Sciences of Ribeirao Preto, University of Sao Paulo, Ribeirao Preto, Brazil; ^6^ Department of General and Specialized Nursing, School of Nursing of Ribeirao Preto, University of Sao Paulo, Ribeirao Preto, Brazil

**Keywords:** coronavirus disease 2019, peripheral immune response, pulmonary inflammation, lung immunopathology, damage tolerance

## Abstract

In the last few months, the coronavirus disease 2019 (COVID-19) pandemic has affected millions of people worldwide and has provoked an exceptional effort from the scientific community to understand the disease. Clinical evidence suggests that severe COVID-19 is associated with both dysregulation of damage tolerance caused by pulmonary immunopathology and high viral load. In this review article, we describe and discuss clinical studies that show advances in the understanding of mild and severe illness and we highlight major points that are critical for improving the comprehension of different clinical outcomes. The understanding of pulmonary immunopathology will contribute to the identification of biomarkers in an attempt to classify mild, moderate, severe and critical COVID-19 illness. The interface of pulmonary immunopathology and the identification of biomarkers are critical for the development of new therapeutic strategies aimed to reduce the systemic and pulmonary hyperinflammation in severe COVID-19.

## Introduction

Between the end of December 2019 and the beginning 2020 a progressive number of deaths due to aggressive pneumonia in the province of Wuhan, China, led to the identification of a new coronavirus, called Severe Acute Respiratory Syndrome Coronavirus 2 (SARS-CoV-2), as the cause of Coronavirus Disease 2019 (COVID-19) (1–3). The infection spread globally becoming a pandemic event in March 2020 (4). COVID-19 can be asymptomatic or symptomatic, which is mainly characterized by dry cough, intense throat pain and fever, which may be accompanied by headache, diarrhea, and mild, moderate, or severe respiratory failure. Around 80% of the people infected with SARS-CoV-2 develop a mild form of COVID-19. The remaining ~20% ​​develop more severe forms of the disease, and require hospitalization, often accompanied by intensive care and mechanical ventilation. Approximately 5% of these patients may progress to sepsis and critical clinical conditions with unfavorable outcomes until death, which could be associated with pulmonary hyperinflammation and respiratory failure (5–7). The severity of COVID-19 is associated with comorbidities, which include senescence, obesity, hypertension, and cardiovascular diseases, and chronic lung diseases (8). The constellation of factors that coordinate the generation of mild, moderate, and severe forms of the disease and how the comorbidities affect the magnitude of inflammation and anti-viral mechanisms that eliminate viral loads are critical aspects for the development of strategies for physicians to face COVID-19.

Many physicians and researchers worldwide have mobilized to study this new disease. After nine months of the pandemic, we see a significant number of scientific studies on different topics, such as those focused on the characterization of hyperinflammation, cytokine storm syndrome, leukocyte subsets, antibody response, viral antigens profiling, laboratorial, and clinical aspects from patients, vaccine development, and drug repurposing. This reflects the efforts of the global scientific community in the search for understanding and solutions for COVID-19.

COVID-19 has fostered a deep reflection, prompting us to share a more comprehensive view about this pandemic. From our perspective, it is possible to develop an efficient pathway to track and control COVID-19. This would involve the articulation of leaders from each country, including leaders from Universities that show a collaborative research tradition, along with the articulation of researchers with common research interests at the national and international levels. Unfortunately, Brazil represents an example that is far from the best practice, where a very feeble interaction has occurred between the government and the scientific community. Brazilian authorities denied or minimized the threat that COVID-19 represents, as recently reported in The Lancet (9, 10). However, Brazilian foundations for research development have encouraged COVID-19 research, and Brazilian scientists have shown important knowledge about the epidemic transmission and evolutionary trajectories of SARS-CoV-2 lineages in Brazil (11) and the epidemiological and clinical characteristics of the COVID-19 epidemic in Brazil (12). Moreover, Brazilian researchers conducted a multicenter, randomized, open-label, three-group, controlled trial involving hospitalized patients at 55 hospitals with suspected or confirmed mild-moderate COVID-19 to evaluate the effect of hydroxychloroquine, alone or with azithromycin, and reported no benefits on the use of both drugs (13). In other investigation from the same group, a randomized, open label, clinical trial at 57 centers was conducted with 397 severe COVID-19 patients. The inclusion of azithromycin to standard of care showed no improvement in clinical outcomes compared to 183 patients to the control group (standard of care, which included hydroxychloroquine) (14).

Considering that we have experienced a pandemic for the first time, the actions of researchers with common investigation objectives are critical worldwide. COVID-19 has affected all countries and ethnicities; therefore we should use it as an example to generate a worldwide research consortium, to encourage scientific cooperation between different research groups with common interests worldwide, promoting internationalization by increasing partnerships, using standardized approaches and methodologies among the different laboratories.

After this brief reflection, in this review, we will address the advances, key points, and critically analyze the comprehension of pulmonary immunopathology in the COVID-19. The understanding of pulmonary immunopathology will contribute to the identification of biomarkers, the other issue that we will address here in an attempt to classify mild, moderate, and severe COVID-19 illness. The broad understanding of immunopathology and the development of biomarkers are both crucial to follow the progression of infection, to predict the disease severity, and choose the best therapy. These topics are critical for the development of new therapeutic strategies aimed to reduce the hyperinflammation in severe COVID-19.

## Innate and Adaptive Immune Response and Inflammation During COVID-19

### Biobanking Molecular Profiles, Transcriptome, and COVID-19

Collaborative efforts have been launched worldwide (COVID-19 host genetics initiative; COVID Human Genetic Effort). Countries with well-established health care policies for biobanking molecular profiles of the population are now benefiting from retrospective studies using genomic data linked to COVID-19 registries. Moreover, hundreds of prospective studies to pursue genetic variants associated with susceptibility or resistance to SARS-CoV-2 infection and its complications are being carried out. A genome-wide association study (GWAS) with 1980 COVID-19 patients from Italy and Spain has found a 3p21.31 gene cluster linked to susceptibility to severe disease (15). Within this genomic cluster, one of the target candidates to be investigated is the SLC6A20 gene that codes amino acid (proline) transporter (SIT1), which interacts with ACE-2 (16), the main cell entry for SARS-CoV-2.

The analysis of 700 lung transcriptome samples of patients with comorbidities associated with COVID-19, such as hypertension, chronic obstructive pulmonary disease (COPD) and diabetes, showed that the expression of ACE-2 receptor was higher in those patients compared to controls (17). ACE-2 was identified as the cell entry receptor for SARS-CoV as well as SARS-CoV-2 (2). Therefore, one of the reasons that comorbidities contribute to the development of severe COVID-19 might be the virus entry facilitated by higher expression of the receptor. However, the inflammatory process that characterizes the comorbidities associated with COVID-19 might also play a role in the pulmonary immunopathology and dysfunctions that affect other organs. Of note, a GWAS combined with a transcriptome-wide association study with 2244 patients with a critical COVID-19 form treated in 208 United Kingdom intensive care units (ICU) indicated that COVID-19 severity and mortality might be related to two genetic mechanisms. The first one is associated with susceptibility to SARS-CoV-2 infection in the early disease stages due to low expression of interferon receptor (*IFNAR2*) and interferon-inducible oligoadenylate synthetase (*OAS*) genes. The second mechanism is related to late disease stages and lung non-homeostatic inflammation associated with upregulation of tyrosine kinase 2 (*TYK2*), dipeptidyl peptidase 9 (*DPP9*), and CC-chemokine receptor 2 (*CCR2*) genes (18).

The comorbidities suggest that COVID-19 is heterogeneous. Besides older age, comorbidities such as hypertension, coronary artery disease, obesity, type II diabetes, chronic kidney disease, and chronic pulmonary disease are risk factors for severe disease (8). In a meta-analysis with seven COVID-19 studies, it was reported that the most prevalent comorbidities were hypertension (21.1%) and diabetes (9.7%), followed by cardiovascular disease (8.4%) and respiratory system disease (1.5%) (19). In a study with 41 patients, 13 (32%) had underlying diseases including diabetes, cardiovascular disease, hypertension or chronic obstructive pulmonary disease (COPD) (20), while the evaluation of 138 patients in another study revealed that 64 (46.4%) had comorbidities (21). Moreover, patients admitted in the ICU had a higher number of comorbidities (72.2%) compared to those with no admission on ICU (37.3%) (21), suggesting that comorbidities represent additional risk factor for the development of severe COVID-19. This highlights that a single type of therapy may not treat the different clinical presentations of COVID-19, and specific treatments might be important for clearance of viral load versus mitigation of hyperinflammation.

Obesity-induced inflammation and metabolic dysregulation could aggravate COVID-19 illness by pathways dependent on the induction of hypercoagulopathy and the infection of adipose tissue (22). The white adipose tissue (WAT) could represent an additional niche for SARS-CoV-2 replication once obesity induces ACE-2 receptor expression on adipocytes. Moreover, obesity generates deposition of WAT on large airway walls inducing an increase of lung wall thickness. Obesity-induced inflammation causes neutrophil influx, pulmonary damage and fibrosis, while lung wall thickness impairs gas exchange (22). Unexpectedly, transcriptional analysis from two COVID-19 patients showed that levels of lung IL-6 mRNA expression, a biomarker associated to COVID-19 hyperinflammation (23), were similar to those in healthy individuals. Despite that, two types of human lung epithelial adenocarcinoma cell (Calu-3 and A549) and normal human bronchial epithelial cell (NHBE) infected with SARS-CoV-2 showed higher levels of IL-6 mRNA in comparison with uninfected cells (24). Certainly, the mechanisms that trigger pulmonary immunopathology during COVID-19 deserve investigation in an attempt to identify different targets to control inflammation.

Given the difficulty of accessing lung samples from individuals with mild disease and considering the limitation of immunological and inflammatory molecules that can be measured in peripheral blood, the evaluation of receptors and mediators associated with the activation of inflammatory response and other biological pathways through transcriptome analysis is an extremely important approach. Transcriptomics is powerful in providing a landscape of molecular mechanisms involved in any condition whether homeostatic or pathological. A valuable number of pioneering studies focused on transcriptomics of systemic and local responses in COVID-19 patients not only has corroborated the immunological findings, but also has shown how complex and diverse the disease might be. Transcriptome data at single-cell resolution of peripheral response by peripheral blood mononuclear cells (PBMC) transcriptional profiling (25, 26) and local response by bronchoalveolar lavage fluid (BALF) transcriptional profiling (27, 28) depicted the role of type I IFN and chemokines signatures, respectively. At the local site, the participation of neutrophils and monocyte-derived macrophages (24, 27–29) has been highlighted in the inflammatory response of severe cases of COVID-19. However, the transcriptional and functional response of peripheral neutrophils in COVID-19 remains to be explored, because the systemic studies so far have employed PBMC rather than total leukocytes.

Pro-inflammatory responses mediated by IL-1β have been featured in severe outcomes of infection, with transcriptional signature observed both at peripheral (26) and lungs (28). Transcriptome analysis of BALF also shed light on physiopathological processes triggered by SARS-CoV-2 infection, unveiling the role of club cells in mucin hypersecretion and ciliary dysfunction in lung epithelial cells can lead to severe COVID-19 (28). Overall, the transcriptome studies have raised convergent observations, but also have shown differences likely due to targeted cell populations and methodological approaches and mainly, because of the small number of analyzed samples. As soon other transcriptome data become available inquiring a plethora of cohorts, more mechanistic insights underlying the disease will be provided, fostering the search for drug repurposing, as well as novel therapies. Importantly, the role of aging and comorbidities should be taken in consideration in such studies, as they can dramatically affect the transcriptional patterns in tissues.

In the last three months, the differences between COVID-19 patients with or without diabetes started to be reported. Eighty COVID-19 patients were classified in diabetes, secondary hyperglycemia or euglycemia groups. The frequency of severe cases was higher in diabetes patients that showed higher concentrations of C-reactive protein (CRP), lactate dehydrogenase (LDH) and IL-6, lower CD4/CD8 cell ratio compared to other two groups. Moreover, diabetes patients presented diverse multifocal features in CT images (30). In other study with 33 diabetes and 37 non-diabetes COVID-19 patients, those diabetes patients showed no differences in IgG, IgM, IgA, C3, and C4 complement proteins, and lymphocytes compared to non-diabetes patients. However, diabetes patients showed higher neutrophil counts, higher CRP, IgE, and IL-6, TNF and IFN-*γ* levels (31). These studies show that inflammation triggered by diabetes courses with worse prognosis, severe COVID-19, and pulmonary damage.

### Peripheral Inflammation and Immune Response

Higher concentrations of IL-2, IL-6, IL-7, IL-10, IFN-γ, G-CSF, TNF, and chemokines such as CCL2/MCP-1, CCL3/MIP-1a, CXCL10/IP-10 were found in the serum of patients compared with healthy donors (20, 32). The same was reported in a study with 187 patients, with 145 survivors and 28 non-survivors: increased concentrations of IL-6 and IL-10 in all patients compared to normal values (33). A comparison performed with 40 patients showed an increase of IL-2, IL-6, IL-10, and IFN-γ in the serum of 13 patients with the severe form of COVID-19 compared to 27 cases of mild disease (34). A study with 102 patients and 45 healthy controls showed differences only in the concentrations of IL-6 and IL-10 in critical patients compared to those with severe and moderate disease (32). This evidence was also reported in the study that described an increase of IL-6 levels in 31 (30.39%) of 102 patients with mild disease, and in 16 (76.19%) of 21 patients with severe disease (35). Both studies suggest that IL-6 and IL-10 are predictive factors of COVID-19 severity and progression. Moreover, the differences among the reported findings might be associated with comorbidities. In addition, the detrimental role of IL-6-IL-6 receptor (IL-6R) axis in the early phase of SARS-CoV-2 infection was not confirmed in a randomized, double-blind, placebo-controlled trial. In this study, the authors reported that the treatment of 161 COVID-19 patients with a single dose (8 mg/kg) of tocilizumab (anti-IL-6R monoclonal antibody) did not prevent death and intubation of patients with moderate disease (36). It would be important to evaluate the tocilizumab treatment in patients with severe and critical COVID-19 illness.

The cytokine storm in the circulation and the diffuse alveolar damage in the pneumonia of severe COVID-19 patients are features shared with Acute Respiratory Distress Syndrome (ARDS) (37). The peripheral hyperinflammation could also be a consequence of secondary haemophagocytic lymphohistiocytosis (sHLH), a rare syndrome that causes immunological hyperactivation, which was previously associated with viral infections, including SARS-CoV-2 (38). sHLH is characterized by high levels of circulating cytokines and chemokines, and by a reduction in CD8^+^ T lymphocytes and NK cells (38, 39). Similarly, severe cases of COVID-19 are accompanied by a reduction in all lymphocyte populations: B, CD4^+^ T, CD8^+^ T, and NK cells, and by neutrophilia (5, 40). Moreover, serum levels of IL-6, IL-10, and TNF were negatively correlated with the number of circulating T cells (41). Considering that circulating human lymphocytes and monocytes are infected by SARS-CoV-2, as reported in two studies that have not been certified by peer review yet (42, 43), the blood circulating leukocytes may have important implications for disease pathogenesis.

An analysis of 452 patients, 286 with severe COVID-19, exhibited a reduction of CD4^+^, CD8^+^ and regulatory T lymphocytes, a reduction of monocytes, eosinophils and basophils, and an increase in the neutrophil-to-lymphocyte ratio (NLR) (44). In an evaluation of 210 patients, 87 developed severe illness and exhibited higher NLR compared to the mild illness group (45). Out of 95 patients, 56 were cured and 39 died, a mortality rate predicted by the NLR (46). Thus, NLR appears to be an early risk factor for severe COVID-19 and should be considered as criteria for classification of severe COVID-19, as well as a criteria for monitoring the treatment (44–46). The increase in the NLR was significantly higher for neutrophils and CD8^+^ T, but not CD4^+^ T lymphocytes, in patients needing ICU compared to non-ICU, suggesting that neutrophil/CD8^+^ T lymphocytes ratio could be a more accurate biomarker for severe COVID-19 (47). However, a high neutrophil/CD4^+^ T lymphocytes ratio was associated with higher incidence of severe disease during admission, and the authors suggest that a reduction in the neutrophil/CD4^+^ T lymphocytes ratio could be a biomarker for virus negative conversion (48). These results were confirmed in a multicenter retrospective cohort with 548 cases with discharged or deceased outcomes from 575 hospitals throughout China: patients were categorized on admission into mild/moderate (345), severe (155) and critical (48) degree (49).

The dynamic changes in hematologic and inflammatory biomarkers in survivors and non-survivors during progression of COVID-19 on admission, hospitalization, and end-hospitalization were used to classify these biomarkers into 3 distinct patterns. The pattern 1, including eosinophils, lymphocytes, and platelets, showed an upward trend in survivors, but a downward trend or kept low in non-survivors. The pattern 2, including neutrophils, NLR, IL-6, procalcitonin, D-dimer, prothrombin time, amyloid A protein, CRP, and ferritin were maintained at lower levels or downward trend in survivors and upward trend or maintained higher levels in non-survivors. Whereas, in the pattern 3, all biomarkers were in normal range and this pattern was associated with survivors. The authors suggested that restored levels of lymphocytes, eosinophils, and platelets could serve as predictors for recovery, whereas progressive increases in neutrophils, basophils, and IL-6 were associated with fatal outcome. In addition, an increase in naive T lymphocytes, and a reduction of memory T lymphocytes, CD4^+^ T cells and regulatory T cells was described in the peripheral blood of patients with the severe COVID-19 (44). Significant changes in peripheral blood parameters associated with different outcomes of COVID-19 patients are summarized in **Table 1**.

**Table 1:** Significant changes in peripheral blood parameters associated with different outcomes of COVID-19 patients.

	Mild *vs* Severe	Mild *vs* Critical	Severe *vs* Critical	Survivor *vs* Non-survival
**D-dimer**	↑ [34; 45; 47; 49]	↑ [49]	–	↑ [33; 46; 49]
**SAA**	↑ [34; 49]	↑ [33]	–	↑ [33; 49]
**CRP**	↑ [34; 44; 45; 47; 49]	↑ [32; 33; 49]	↑ [32]	↑ [33; 46; 49]
**IL-6**	↑ [32; 34; 35; 44; 45]	↑ [32]	↑ [32]	↑ [33; 46; 49]
**IL-10**	↑ [34; 35; 44]	↑ [32; 33]	↑ [32]	↑ [33; 46]
**Neutrophil**	↑ [34; 44; 45; 47; 49]	↑ [49]	↑ [49]	↑ [33; 46; 49]
**NLR**	↑ [44; 45; 47; 49]	↑ [49]	↑ [49]	↑ [49]
**Lymphocytes**	↓ [34; 41; 44; 45; 47; 49]	↓ [33; 41; 49]	–	↓ [33; 46; 49]
**CD4^+^ cells**	↓ [35; 41; 44; 45; 49]	↓ [33; 41]	–	↓ [33; 46; 49]
**CD8^+^ cells**	↓ [34; 35; 41; 45; 47; 49]	↓ [33; 41]	–	↓ [33; 46; 47]
**NK cells**	–	↓ [33]	–	↓ [33; 46]

SAA, Serum amyloid A; CRP, C-reactive protein; IL-6, Interleukin 6; IL-10, Interleukin 10; NLR, neutrophil-to-lymphocyte ratio; CD4, cluster of differentiation 4 – T helper cell; CD8, cluster of differentiation 8 – cytotoxic T cell; NK cells, Natural Killer cells. ↑ Increased or ↓ decreased in COVID-19 patients.

A study that evaluated circulating T lymphocytes in 4 elderly patients showed differences in the populations of CD4^+^ and CD8^+^ T cells expressing markers associated with activation, immune memory and suppression, such as PD-1 (50). However, this analysis was not accompanied by a description of the outcome of COVID-19 in those individuals. Still, peripheral lymphopenia was followed by markers that characterize lymphocyte senescence/exhaustion, such as an increase of NKG2, CD57, or PD-1 expression on CD8^+^ T lymphocytes and NK cells (51, 52). Twenty eight studies were reviewed involving 3939 COVID-19 patients in China, and the authors described the reduction of lymphocyte subsets and neutrophilia followed by increased levels of cytokines, specifically IL-6 (40). Antagonists of IL-6 receptor have shown promising results in clinical trials, and antibodies against IL-1, IL-1R, IL-8, TNF, GM-CSF, GM-CSF receptor, IFN-γ, and IL-17 are under evaluation (40). Therefore, several lines of evidence indicate that a cytokine storm and neutrophilia, which are hallmarks of activation of innate response and reveal dysfunction of the immune system, induce down-regulation of the adaptive immune responses that are crucial for infection control. In this scenario, high levels of IL-6 negatively regulate perforin and granzyme B levels, one of the reasons that may affect the activity of NK cells (53). The detrimental role of neutrophils was described in a study with 32 patients showing that the concentrations of neutrophil extracellular traps (NETs) were higher in plasma and tracheal aspirate from severe COVID-19 patients compared to the controls. The authors showed that NETs induced the death of A549 airway epithelial cell line (54). It will be important to assess the effect of NETs on lymphocytes. In this context, COVID-19 patients displaying alterations in the T cell compartment that leans toward the functional phenotype Th17 could favor the occurrence of hyperinflammation by the exacerbated release of IL-17, a potent chemoattract/activator for human neutrophils (51).

The early induction of the adaptive immune response seems to be important for the best prognosis of the disease, since a case-report study described that the recovery of an infected woman in China was associated with an increase in antibody-producing cells and follicular CD4^+^ T cells, accompanied by increased levels of IgM and of IgG in the peripheral blood on days 7 and 8 of symptoms. Such parameters remained high on day 20, when the patient was already recovered. The concentrations of inflammatory cytokines and chemokines were reduced and the patient did not use any medication until recovery (55). It will be essential to assess whether this type of immune response indeed characterizes individuals with mild disease or whether other biomarkers could more accurately define the stratification between mild and severe forms. The characterization of leukocyte subsets and phenotypes, and functional activity of these cells in the peripheral blood of asymptomatic individuals, as those in close contact with patients, may provide information associated with the protective immune response, while the study of oligo-symptomatic patients in different cohorts will provide the dimensions of a balanced immune response that reflects pathogen resistance and damage tolerance. A Chinese study reported the evaluation of 25 asymptomatic COVID-19 patients, and showed that asymptomatic patients exhibited normal clinical indicators characterized by increased number of T, B, and NK cells, lower LDH compared to 27 symptomatic patients, which exhibited liver damage. Asymptomatic patients showed a faster viral clearance, although they may represent a greater risk of virus transmission (56). The increase of CD8^+^ T cells might be a biomarker for the efficacy of treatment for COVID-19, as patients who had a positive response for the treatment exhibited increases of those cells (57). Lastly, the evaluation of functional activity of lymphocytes in the peripheral blood of recovered individuals could make a huge contribution in attempts to characterize the protective immune response against SARS-CoV-2 infection.

There are contradictory indications in the literature about the role of lymphocytes in the immunopathology of COVID-19, since it has been reported that SARS-CoV-2 infection could provoke an immunological hyperactivation and accumulation of T lymphocytes in the lungs of patients with severe illness (38, 58) or T cell exhaustion/senescence (41, 51, 52, 59).

In this scenario, two very elegant studies appear to be the first to show the stratification of different COVID-19 clinical forms associated with different immunotypes within the same clinical form and depending on the phase of disease. These two studies used different approaches to perform longitudinal evaluation and analyze immune and clinical features. On study involved a longitudinal evaluation from 1 to 25 days from symptoms onset with 113 COVID-19 patients with moderate (non-ICU) and severe (ICU) disease. This study indicated that moderate cases were grouped in a cluster 1 that characterizes low expression of inflammatory cytokines and an increase in gene expression associated with growth factors or tissue repair genes. By contrast, severe cases were grouped in clusters 2 and 3 that characterize inflammatory cytokines and type 2 cytokines. Cluster 3 represented a more intense pro-inflammatory cytokine signature, worse disease and eventually death. In addition, this study indicated that although COVID-19 patients with severe illness in the first days from symptoms onset exhibited augment of IL-6 and IL-10, at late days from symptoms onset, they showed increased levels of IFN-α and IFN- λ, IL-17, IL-22, eotaxin, IL-13, and a reduction of IL-6. Moreover, severe patients displayed an increase of monocytes with down-regulation of HLA-DR (60). This study reports the novel finding that in the initial or in the later symptoms onset, patients with severe illness show different status of immune activation, defined as immunotypes.

In a study with 125 patients hospitalized at the University of Pennsylvania, Mathew and coworkers confirmed what has been already reported, i.e., patients were older age than recovery patients and healthy controls, and cardiovascular disease was the mostly associated comorbidity to COVID-19. The great majority of patients exhibited increased CRP, high LDH, D-dimer, and ferritin. Lymphopenia was reported in approximately half of the patients and high levels of IL-6 were detected in 31 from 46 subjects evaluated (61). However, an amazing aspect to this study is the elegant way in which it was shown that different immunotypes occur in hospitalized patients and the differences in the time-dependent trajectory of severe disease, using a multidimensional integrated analysis with several immunological and clinical parameters. Immunotype 1, identified in more severe cases, was characterized by intense activation and proliferation of CD4^+^ T cells, activation of CD8^+^ T cells, presence of exhausted CD8^+^ T cells and low frequency of circulating follicular helper cells (cTfh). Immunotype 2 was defined by the presence of T bet^bright^ CD8^+^ cells, reduced activation of CD4^+^ T cells compared to immunotype 1, and presence of memory B cells and proliferating plasmablasts (PB). Immunotype 3, which was identified in ~20% of patients, showed minimal activation of T cells, suggesting fails in the activation of immune response. The longitudinal analysis of severe cases was associated with immunotype 1 (61), suggesting an immunopathology induced by unbalanced adaptive immune response.

Proteomics and metabolomics analysis revealed with 93.5% accuracy that serum contains 22 proteins and seven metabolites that may help to classify severe COVID-19. Besides CRP, other acute phase proteins including SAA1 and SAA2, SERPINA3, VCAM-1, C6 protein of complement system, and steroid hormones were increased, while sphingolipids, fatty acids, among other lipid metabolites were decreased (62).

In this context, the field needs to further research larger cohorts that compare the mild, moderate, severe and critical forms of the disease. At the moment there are a small amount of evidence in this regard, each one evaluating different parameters of immune response and inflammation.

## Lessons From Coronavirus Infections

During epidemics resulting from coronavirus infections in 2002/2003 (Severe Acute Respiratory Syndrome Coronavirus - SARS-CoV) and 2012/2013 (Middle East Respiratory Syndrome - MERS), respectively, researchers suggested, based on lung histopathological analysis from individuals who died of pulmonary disease, that the progressive and late form of SARS-CoV disease was more associated with tissue damage caused by immunopathology than with viral load (63). Although SARS-CoV and MERS were more lethal than COVID-19, the hallmark of this last is its higher transmissibility by airways. The oro-fecal transmission was not confirmed yet despite the detection of viral particles in feces (64). Even though high serum levels of IgM against SARS-CoV-2 and inflammation/tissue injury biomarkers (IL-6, CRP, and LDH) were reported to a neonate born to an infected mother, there is still no robust evidence that confirms potential vertical transmission of this coronavirus (65–67).

As SARS-CoV and MERS, COVID-19 causes a huge inflammation, described as a cytokine storm, and diffuse alveolar damage that characterize the severe form of illness (40). Pulmonary pathology induces respiratory failure, which is followed by damage in other organs, such as kidneys, liver and cardiovascular and nervous systems. The fact that SARS-CoV-2 infection causes intense pathological inflammation shows that surviving the infection is more a matter of tolerating lung damage (damage tolerance) than properly controlling viral load (pathogen tolerance), as already described (68, 69). Therefore, although the development of vaccines against SARS-CoV-2 is crucial for the protection of population worldwide and the development of new drugs or drug repurposing is critical for viral clearance, the understanding of pulmonary immunopathology during COVID-19 is the key for treatment aimed to reduce inflammation in the severe forms of the disease. Additionally, this knowledge is of great value for the development of safe vaccines that avoid clinical severity to subsequent infections and potential side effects of convalescent plasma therapy for COVID-19 (70–72). New perspectives for the treatment of pneumonia involve host-directed therapies and should include therapeutic measures aimed at damage tolerance mechanisms (69). The concept of host-directed therapies is particularly important for COVID-19, which shows heterogeneous clinical forms and requires the study of intrinsic factors as the genetic background, and extrinsic factors, associated with environmental factors and comorbidities.

## Pulmonary Inflammation and Immunopathology

Considering that most studies published to date evaluated inflammatory mediators and leukocytes in peripheral blood and that immune response may be under compartmentalization, what is found in the periphery sites does not necessarily occur in the lungs. The understanding of the immunological and inflammatory response at the first and main site of the disease is fundamental for the identification of mediators, receptors and cells that cause dysfunction of immune response and how such dysfunction affects the progression of disease. The focus on pathological immune response that cause hyperinflammation and, as consequence, ARDS, will probably determine the design of therapies that may be efficient to down regulate the pulmonary inflammation.

In a recent review article, 23 studies were reported with post-mortem patients and evaluation of pulmonary pathology (73). The first of them, published in February, described a diffuse alveolar damage in a 50 year-old male, similar to those already described for MERS and SARS-CoV (74). The diffuse alveolar damage and histological findings compatible with ARDS were also reported in subsequent studies with deceased COVID-19 patients (75, 76). However, while one study showed extensive lymphocyte infiltration in the lungs (74), the other study that evaluated 4 COVID-19 patients reported only neutrophilic infiltration (75). The number of articles with post-mortem and pulmonary pathology, increased from April, and describe endotheliitis, coagulopathy and pulmonary emboli associated with influx of neutrophils in the alveolar space and trachea mucosal, presence of NETs and activation of complement system. An example is the post-mortem examination of 21 COVID-19 patients from Switzerland that described a diffuse alveolar damage accompanied by capillary congestion and presence of microthrombi. Bronchopneumonia was reported in 10 patients, 4 with pulmonary embolism, 3 with alveolar haemorrhage and 1 with vasculitis. The disease preferentially affected males with cardiovascular comorbidities and individuals with blood group A (77). Although it was described that co-localization occurred between viral particles and endothelial cells in different organs as lungs, kidneys and heart (78), another study with 11 post-mortem individuals showed that SARS-CoV-2 was detected in different organs such as intestine, liver and kidneys besides lungs, but the inflammation was focused on lungs and secondary lymphoid organs (7). Interestingly, there was no association between viral loads or the presence of viral antigens and inflammation. Although the lung inflammation is a consequence of SARS-CoV-2 infection, the hyperinflammation that induces immunopathology and respiratory failure may be consequence of dysfunction in the activation of immune response caused by alveolar cell death and alveolar congestion that reduces oxygen flow.

A very interesting and elucidating study that has not been certified by peer review yet reported analysis of lung autopsy material from 24 deceased COVID-19 patients (14 men and 10 women; mean age of 62.5 years old) collected in two institutions of the United States of America. First, they confirmed the data from different studies that COVID-19 is more prevalent in men and in older adults. The patients were classified as high or low viral RNA load, with the predominance of intracellular viral RNA in pneumocytes associated with extracellular viral RNA detection in hyaline membranes (79). In contrast to the results reported by Dorward and coworkers (7), a high viral RNA load was associated with high expression of IFN pathway, endothelial and wound healing genes, which corroborate the findings of lung damage characterized by exudative diffuse alveolar damage, fibrosis, infiltration of macrophages, monocytes, T cells and NK cells in areas positive or negative for SARS-CoV-2, and high expression of CTLA-4, PD-L1, and IDO. The low viral RNA load was associated with the presence of M0 and M2 macrophages, wound healing and presence of more intact pneumocytes (79). Dorward and coworkers also reported the decreased counts of resident macrophages in bronchoalveolar lavage fluid and pulmonary vasculitis characterized by the predominance of myeloid cells and monocytes; however, vasculitis was not necessarily associated with viral S protein on endothelial cells. Myeloid cells and CD4^+^ and CD8^+^ cells (in a lower extent) accumulated in the pulmonary parenchyma (7). Recently, two immunopathological profiles associated with signature of high or low interferon-stimulated genes (ISG^high^ and ISG^low^, respectively) were described in the lungs of deceased COVID-19 patients. Patients with ISG^high^ signature remained in the hospital for a shorter period than patients with ISG^low^ signature. Lung samples from ISG^high^ patients exhibited higher viral load and higher production of inflammatory cytokines and chemokines compared to lung samples of ISG^low^ patients, which showed higher number of CD4^+^, CD8^+^, CD20^+^ (B cells), CD163^+^ (monocytes), and CD64^+^ (macrophages) cells, complement deposition, diffuse alveolar damage, and coagulopathy, characterizing an immunopathology profile (80). The authors describe that the variability in the profiles is due to initial infectious dose and genetic background to generate an immune response.

To gain insights about the mechanisms that trigger SARS-CoV-2-induced inflammation, Li and coworkers showed that viral infection of Calu-3 cells induced apoptosis dependent on caspase 8 and production of IL-7, IL-8, TNF, CXCL-10, and CCL5. They also showed the induction of apoptosis, necroptosis and inflammation in the lungs using the ACE-2 transgenic mouse model. Furthermore, the analysis of lung sections from deceased COVID-19 patients confirmed the presence of apoptosis, necroptosis, necrotic cell debris and interstitial fibrosis (81). Therefore, two types of cell death and caspase-8-dependent inflammation may induce pulmonary immunopathology. Using a proteomic approach combined with bioinformatics analysis, Leng and coworkers reported the pathogenesis of SARS-CoV-2 at the molecular level using lung tissue samples from four COVID-19 patients. For proteomics analysis, lung samples were obtained from two elderly patients, one male and one female. From 641 proteins differentially expressed, 222 proteins were upregulated and 419 were downregulated compared with control (paracancerous tissue of lung cancer with no COVID-19). Most of proteins, up or downregulated, were localized in the nucleus. RIG-I pathway was activated. Downregulated proteins were found to be associated with endoplasmatic reticulum, lysosomes, ribosomes, cytoskeleton, and cell membrane. TLR4, CD40, IL-6, IL-8, TNF, IFN-α, ICAM1, and CXCL12, which are associated with NF-kB pathways, and TRAF protein family members were upregulated in the lungs of COVID-19 patients. Angiogenesis pathway, adhesion, and interaction with the extracellular matrix receptors were decreased, while activation of coagulation cascade, MMP2, MMP8 and CTSS cathepsin were upregulated (82). Although with a small number of lung samples from patients evaluated, this is a very interesting study because it identifies at the site of inflammation, possible molecular targets to be investigated as therapy to balance immunopathology.

The mechanisms underlying lymphopenia and lymphocyte exhaustion in peripheral blood, as well as lymphocyte accumulation in the lungs remain to be investigated. The findings reported herein clearly depict the heterogeneous immune response in the later phase of critical disease and highlight the efforts to design different immune therapies. Some characteristics mentioned above are summarized in **Table 2**.

**Table 2:** Lung immunopathological characteristics of COVID-19 patients.

	Lung	BALF
**Diffuse alveolar damage**	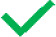	
	[7; 74; 75; 76; 77; 80]	
**Pulmonary or intra-alveolar hemorrhages**	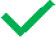	
	[75; 77; 80]	
**Desquamation of pneumocytes**	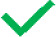	
	[74; 75; 73; 80]	
**Hyaline membrane formation**	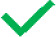	
	[74; 75; 76; 77; 82]	
**Vascular congestion**	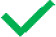	
	[75; 77; 78]	
**Pulmonary or alveolar thrombi**	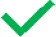	
	[7; 77]	
**Type II pneumocyte hyperplasia**	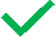	
	[75; 80]	
**Atypical enlarged pneumocytes**	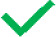	
	[74; 82]	
**Mononuclear inflammatory infiltrates**	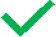	
	[7; 74; 75; 76; 78; 80]	
**Lymphocytes infiltration**	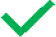	
	[74; 75; 77; 80; 81]	
**CD8^+^ T cell infiltration**	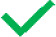	
	[7; 76; 80]	
**CD20^+^ B cell infiltration**	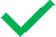	
	[7; 76; 80]	
**Monocytes and macrophages infiltration**	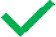	
	[7; 76; 79; 80; 81]	
**Neutrophilic infiltration**	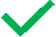	
	[7; 75; 77; 80]	
**Apoptotic bodies**	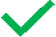	
	[78]	
**Intra-alveolar viral cytopathic-like changes**	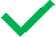	
	[74]	
**Interferon gamma response genes**	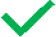	
	[79; 80]	
**Mucin genes**		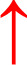
		[28]
**IL-1β**	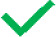	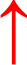
	[81]	[28]
**Chemokines**	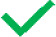	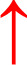
	[80; 81; 82]	[28; 29]
**IL-10 and TGF-β**		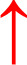
		[29]
**Neutrophil function**		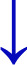
		[29]

BALF, Bronchoalveolar lavage fluid; IL-1β, Interleukin 1 beta; CCL, chemokine (C-C motif) ligand; MCP-1, monocyte chemoattractant protein-1; MIP, macrophage inflammatory protein 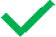. Observed in lung sample; 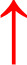 Increased or 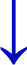 decreased in BALF of COVID-19 patients.

Although these studies (7, 78, 79) show an evident progress forward to characterization of pulmonary pathology, much remains to be learned about damage tolerance for COVID-19 to translate this knowledge into clinic application. In this sense, the functional activity of T cell subsets in the lungs was not yet reported, but this information will be important to characterize the scenario of inflammation in the lung microenvironment. Other key point to address is the quantification of regulatory T cells, their influx and suppressive activity in the pulmonary parenchyma of severe patients because the balance of effector T cells and regulatory T cells is critical for damage tolerance.

This issue brings us back to the cytokine storm. The overproduction of IL-2, IFN-γ, IL-6, and IL-10 in the peripheral blood would also be the hallmark within lungs? If this clinical evidence in blood finds a confirmation in the lungs, would the increase in IL-2 concentrations in the lungs be associated with the expansion of regulatory T cells? In this context, it would be interesting to assess the ratio of CD4^+^ T and regulatory T cells, although both are reduced in the circulation of patients with severe forms of the disease. Therefore, could a reduction of T lymphocytes and regulatory T cells in the blood represent their accumulation in the lungs? Considering the lymphopenia and expression of exhaustion markers on lymphocytes in severe illness, could this reflect a lower influx within the lungs, and as consequence, a low strength for an effective adaptive immune response? The case-report with an infected woman in China, previously mentioned here, describes that during the symptomatic phase, lymphocyte, neutrophil and NK cell counts in the serum were comparable to basal levels. However, the patient exhibited a reduction in the number of CD14^+^CD16^+^ monocytes (55). A reduction of CD14^+^CD16^+^ monocytes could be an indicative for the monocyte migration to the lungs. Considering the participation of reticulo-endothelial system and accumulation of macrophages and monocytes in the pulmonary parenchyma of post-mortem patients, and the congestion of myeloid cells in the pulmonary arteries (7), it is also critical to evaluate the phenotype of monocytes and macrophages as well as their functional activity in the lungs of patients with severe COVID-19. The density of monocytes/macrophages and CD4^+^ and CD8^+^ T lymphocytes in the lungs of patients with severe COVID-19 as well as their functional role are key factors to predict the extension of immunopathology. For sure, comorbidities play a role on that because obesity was associated with a high peripheral neutrophil/CD8^+^ T lymphocyte ratio, an indicative of poor prognosis in patients with COVID-19 (47).

In addition, obesity might also play a role in the coagulopathy described in severe form of COVID-19 because it induces overproduction of procoagulant factors that induce thrombosis (22). Interestingly, IL-6 may participate in the induction of coagulation cascades (83) and levels of IL-6, IL-10 and TNF were negatively correlated with the number of circulating T cells (41). Moreover, severe COVID-19 patients exhibited increased platelet activation and platelet-monocytes aggregates compared with mild COVID-19 patients. Platelet activation and expression of tissue factor by monocytes were associated with increased fibrinogen and D-dimer, markers of coagulation dysfunction, in severe COVID-19 patients (84). It remains to be investigated whether other COVID-19-associated comorbidities have similar effect on pulmonary pathology. This understanding is critical for the development of host-directed therapies considering that infection by SARS-CoV-2 results in different clinical outcomes.

This rationale prompts us to consider the initial steps of SARS-CoV-2 infection. To gain insights into early infectivity and disease pathogenesis to SARS-CoV-2, Hou and coworkers used GFP reporter virus to characterize virus tropism and reported that a higher nasal expression of ACE-2 compared to bronchial airway epithelia, along with high infectivity of nasal epithelium followed by reduction in bronchioles and alveoli (85). The high expression of ACE-2 and transmembrane protease serine 2 (TMPRSS2) in nasal epithelium compared to lower airways suggest that therapeutic interventions must also consider nasal cavity. In lung autopsies, the authors found that ciliated cells were infected, but not mucus secretory club cells, despite ACE-2 and TMPRSS2 expression (85). Therefore, it remains to be investigated other factors that mediate infection in the lower airways. After infection mediated by ACE-2 receptor expressed on type II alveolar epithelial cells, the lysis of epithelial cells generates the release of DAMPs (danger associated molecular patterns), that are recognized by PRRs (pattern recognition receptors) and perpetuates cytokine release (37) and the recruitment of neutrophils and monocytes (7). IL-18 was correlated with poor outcome of disease and NLRP3 appears to be increased in the lung autopsies of deceased patients (86). However, the treatment of SARS-CoV2-infected Calu-3 cells with caspase-1 or NLRP3 inhibitors did not affect the secretion of IL-1β, suggesting that caspase-8-induced inflammation mediates the production of IL-1β (81). The fact is that we have still a very limited comprehension about initial steps that generate the innate response (87). Initial events may involve also the secretion of type I IFN by most of cells and type III IFN predominantly by epithelial cells during viral airway infections (88). It is been established that SARS-CoV-2 impairs the production of type I and type III IFNs by human epithelial cell lineage. Moreover, normal human bronchial epithelial cells produce no detectable level of type I and type III IFNs and induce limited subsets of ISG, and post-mortem lung samples from COVID-19 patients revealed also no detectable type I or type III IFNs (24). However, epithelial cells stimulated with type I IFN showed an increased expression of ACE-2, which could contribute for viral invasion (89). Post-mortem lung samples from COVID-19 patients showed a robust chemokine gene expression that induces the recruitment of inflammatory monocytes, as previously described in a mouse model for SARS-CoV (90). Monocytes and macrophages express also ACE-2 receptor and induce inflammation (91). Although there is a delay and low levels of secreted type I IFN, ISG are induced and the viral replication is moderate *in vivo*, in a ferret model (24). Although IFN-α has been associated with a protective effect in SARS-CoV infection (92), these mediators may play a pathological role because high levels of them were correlated with sequelae (93). As mentioned above, ISG^high^ signatures in the lungs of deceased COVID-19 patients were associated with high viral load and more severe disease, although both ISG^high^ and ISG^low^ lung signatures were fatal in COVID-19 (80). Furthermore, as also pointed out previously here, a genetic mechanism associated with susceptibility to SARS-CoV-2 infection in the early disease stages is the low expression of interferon receptor (*IFNAR2*) and interferon-inducible oligoadenylate synthetase (*OAS*) genes (15). Therefore, the role of type I IFN appears to be time-dependent and concentration-dependent: protective at moderate levels in the early disease and detrimental at high levels in the progressive disease. Interestingly, the expression of type I IFN in the early disease could be a factor associated with lower susceptibility of female compared to male to COVID-19, considering that the type I IFN is more efficiently produced by plasmacytoid dendritic cells (pDC) in female. Epigenetic modification of X-linked genes would induce higher type I IFN production by female pDC, IFN-α-dependent B cell activation and antibodies secretion associated with viral clearance (94). In addition, Zhou and coworkers showed that although patients with acute severe illness generated neutralizing antibodies, they exhibited a weak specific CD8^+^ T cell response, an increase of conventional DC:pDC ratio and an impairment of DC activation (95).

Therefore, the dysfunction in the innate response with improper recruitment of myeloid cells (neutrophils and monocytes) suggests that treatments for COVID-19 need be driven to controlling inflammation (24). Soluble CD14, a classical marker of monocytes, is increased in the plasma of COVID-19 patients and is associated with severity of disease (37). Considering that CD14-deficient mice infected by influenza A virus did not develop inflammatory response, CD14 could be a target focused on blocking steps in innate immunity to mitigate pulmonary immunopathology (37). To understand the protective or pathological role of monocytes and the magnitude of monocyte influx to the lungs, it will be important to evaluate the severe cases of COVID-19 associated with different comorbidities, which contribute for the diverse degree of immunopathology. Moreover, it will be important to understand the participation of epithelial cell in the generation of inflammation, which is the first entrance door for SARS-CoV-2.

**Figure 1:**
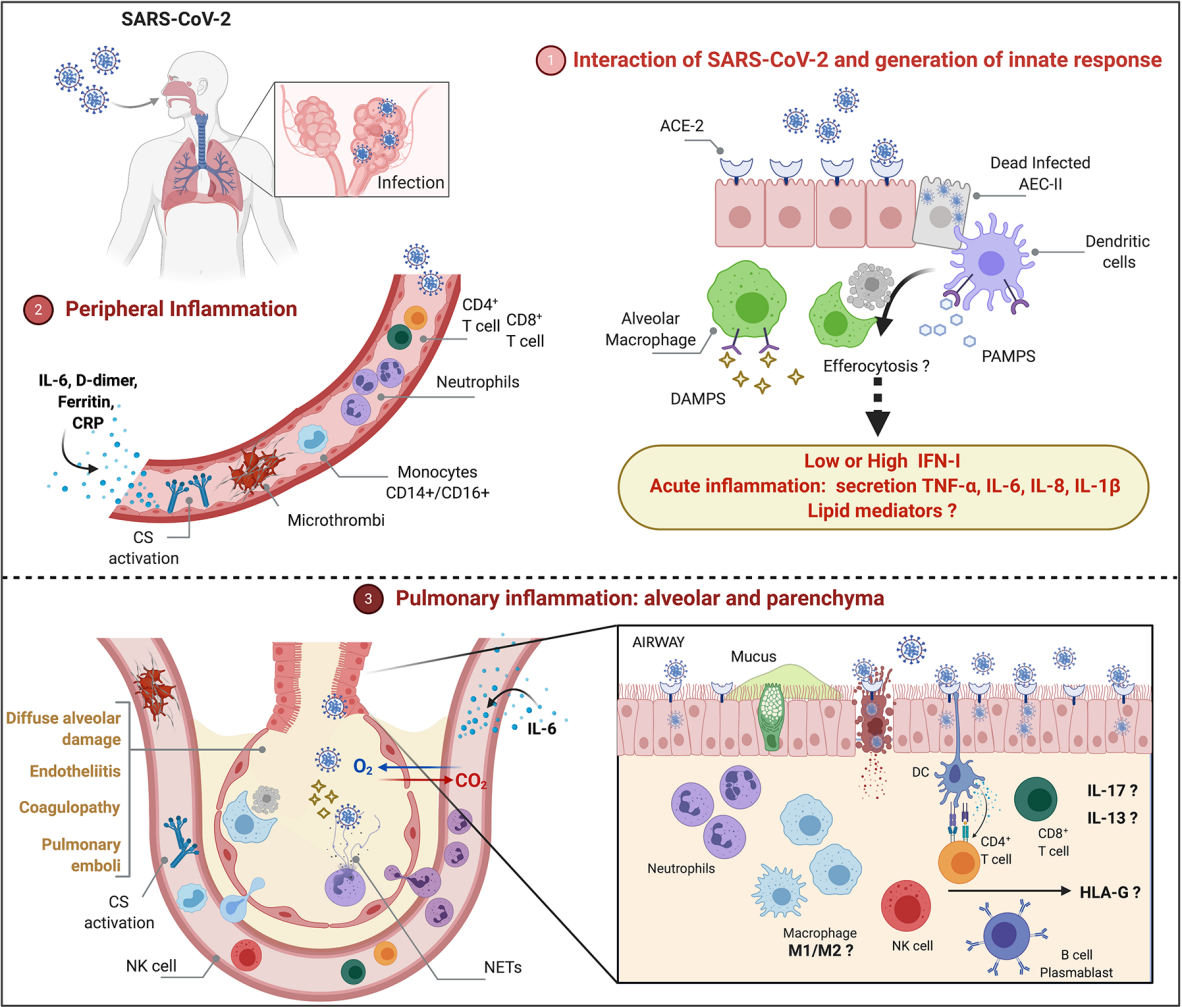
Peripheral and pulmonary inflammation and immunopathology during severe COVID-19 (1). SARS-CoV-2 infects type II alveolar epithelial cells (AEC-II) that express ACE-2 receptor. Cytolysis of infected AEC-II release DAMPs and PAMPs that may contribute for activation of innate response and induction of acute inflammation. Although SARS-CoV-2 inhibits type I-IFN (IFN-I)-induced cell activation pathways, longitudinal evaluation of patients with severe illness show increased IFN-I that may play a pathological role. Functional activation of alveolar macrophages and dendritic cells, efferocytosis and lipid mediators remain to be investigated (2). Peripheral inflammation is characterized by neutrophilia, lymphopenia (CD4^+^ T, CD8^+^ T, regulatory T, NK cells), increased monocytes (CD14^+^CD16^+^), activation of complement system (CS), increased levels of IL-6, C-reactive protein (CRP), D-dimer and ferritin (3). Severe COVID-19 courses with diffuse alveolar damage, endotheliitis, coagulopathy, and pulmonary emboli associated with neutrophilic/myeloid inflammation and lymphocyte inflammation in lower extent, increased levels of IL-13 and IL-17, accumulation of NETs. The role of M1/M2 macrophages as well as the participation of sex steroid hormones and HLA-G in the immunopathology remains to be investigated.

Because COVID-19 is heterogeneous and pulmonary immunopathology is multifactorial, it will be important to address also the role of steroid hormones, lipid mediators and HLA-G protein in COVID-19. **Figure 1** depicts cells and molecules/mediators that participate of peripheral and pulmonary inflammation and immunopathology during COVID-19.

## Sex Steroid Hormones and COVID-19

The communication between the endocrine and immune systems is fundamental for host homeostasis (96). This interaction depends on mediators such as cytokines and lipid-derived steroid hormones, which may be produced by leukocytes or be influenced by them. Otherwise, classical hormones released by endocrine glands may regulate the immune response and the consequent tissue damage in uncontrolled inflammatory processes (97).

The gonads and adrenal glands produce sexual hormones such as androgens that together with estrogens drive the sexual dimorphism in men and women. Androgens and estrogens activate specific receptors in leukocytes, thus influencing immune function and susceptibility to infection or inflammation (98). There are clear differences between the immunological status of males and females, as observed by the incidence, susceptibility and outcome of diseases in which sexual hormones modulate the patient’s immunity (99–101). In accordance, estrogens play a fundamental role in numerous events in the human physiology and diseases, as observed by the higher incidence of autoimmunity in women compared to men (102–104). In contrast, the androgen testosterone, mainly produced by males, negatively regulates the cytokine BAFF that is an essential factor for B lymphocytes activation and antibody production in autoimmune diseases (98).

Earlier studies from the 1960s decade suggested the increased capacity of women to produce antibodies (100), which are findings that could be related to recent data showing augmented immune reactivity of females as well as their resistance to infectious diseases, including those caused by respiratory viruses such as severe acute respiratory syndrome (SARS) (105), Middle East Respiratory Syndrome (MERS) (106) and influenza (107, 108). Interestingly, while this viral infection results in a more severe disease in men, with increased hospitalization occurrences (108, 109), women also produce higher levels of antibodies in response to influenza vaccination (110). Then, the sex steroid hormones may contribute to the disease worsening or protection, depending on the patient’s disorder and immune-endocrine status.

Despite hormones, various hypotheses have been proposed to explain the sex differences in the COVID-19 outcome, including genetics, immune responses, environmental and cultural factors such as smoking, as well as working routine (111). Men are at an increased risk for SARS-CoV2-induced complications (112), with notable sex differences in the incidence, severity and fatality of this viral disease (113, 114). Indeed, women exhibit reduced incidence and morbidity rates of COVID-19 (113).

The clear genetic discrepancy between males and females potentially contribute to the disease outcome (115). The presence of a single X chromosome in men (in contrast to XX in women) could predispose to reduced alternative mechanisms for protection upon exposure to the virus. Notably, ACE-2 gene is located on X chromosomes and is affected by androgens (116–119), thus accounting for a higher susceptibility to infection in men. On the contrary, estrogen can down regulate the expression of ACE-2 in differentiated airway epithelial cells (120). Furthermore, since the androgen receptor (AR) gene is also located on the X chromosome, the sensitivity to hormones such as testosterone could represent an important determinant factor for the severity of COVID-19 in men (121).

SARS-CoV-2 infects the cells by the binding of its spike protein to the cellular ACE-2 receptor. The TMPRSS2, whose activation is dependent on androgens, primes spike protein for virus entry into the host’s cells and interact with ACE-2 receptors, facilitating viral entry (122). It is of note that the transcription of TMPRSS2 gene depends on AR and increases after exposure to androgens (123, 124). In agreement, there is up-regulation of TMPRSS2 mRNA upon activation of AR (125), while androgen deprivation therapy (ADT) suppresses this axis (126, 127). Moreover, the *in vitro* inhibition of TMPRSS2 can prevent SARS-CoV-2 infection of cultured cells (122). Altogether, this evidence suggest that the elevated expression of TMPRSS2 induced by androgens could be one of the explanations for the increased male susceptibility to COVID-19 and the milder disease in children who still do not have high expression of AR (128).

As discussed above, one of the reasons for such sex dichotomy in COVID-19 could be the difference between the immune response of males and females (129, 130). Higher levels of estrogens are related to stronger reactions that facilitate the pathogen clearance, while testosterone usually inhibits the immune processes, thus supporting the overall assumption that men have reduced autoimmunity as well as attenuated responses against infectious agents. Indeed, the androgen’s effects on innate and adaptive immune responses depends on the hormone signaling through the AR (98, 131–133). A recent study found that critically ill patients with COVID-19 had lower amounts of circulating and free testosterone compared to subjects in a more stable condition, indicating that this androgen could predict the worst disease outcome (134). In line with that, a drug screening study confirmed the capacity of a 5-alpha reductase inhibitor, which prevent the conversion of testosterone into the potent dihydrotestosterone, to reduce ACE-2 levels in cardiac and human alveolar epithelial cells treated *in vitro* with this drug. Patient data also showed that altered androgen conditions are associated to severe COVID-19 complications as well as cardiac injury, thus clarifying some important mechanisms that could lead to augmented men’s susceptibility to this disease (135). Furthermore, besides ACE-2 and AR, other genes related to the immune response are also located on the X chromosomes (136, 137) and could potentially interfere in the virus elimination or inflammation control. Indeed, males had increased IL-8, IL-18, CCL5, and prominent non-classical monocytes, while female patients presented stronger T cell activation, compared to men (138).

Hence, considering that sex hormones such as estrogens and androgens may differentially modulate the inflammatory responses, it is plausible to assume that these steroid immune-endocrine mediators may interfere in the anti-viral immunity and the outcome of COVID-19 in both men and women.

## Lipid Mediators and COVID-19

Lipid mediators, such as leukotrienes (LT) and prostaglandins (PGs) derived from arachidonic acid (AA) metabolism (139, 140), are involved in the induction and in the regulation of immune responses in bacteria, fungi, and parasites infections, as well as in envenomation (141–147). Although LT are considered deleterious, as cysteinyl leukotriene (CysLT) induces muscle contraction when released in upper bronchoalveolar space inducing bronchospasms (148), LTB_4_ has been considerate beneficial to the control of infections, especially in the lungs. LTB_4_ increases phagocytosis and microbial killing (149–152), induces the production of antimicrobial peptides (153), decreases IL-1β production (144). Moreover, pharmacological or genetic inhibition of LTB_4_ depresses immune response against bacteria, fungi, and parasites (142, 149, 152, 154), and LTB_4_ administration improves survival in fungal and bacterial infection (153, 155).

The participation of LT has been also described in viral infection (156). Bronchial epithelial cells infected with respiratory syncytial virus produce LT (157), suggesting a role for 5-LO-derived compound in viral infections. Moreover, experimental models in mice validated the protective role of LTB_4_ as antiviral mediator. The investigators showed that LTB_4_, *via* nucleotide-binging oligomerization domain 2 (NOD2) pathway enhances the anti-influenza A response, and that the treatment of influenza A-infected mice with LTB_4_ increased the release of IFN-β, IL-6, and TNF in the lungs, ameliorating the innate response and increasing mice survival (158). We have previously showed that the increase in circulating LTB_4_ and CysLT is associated with the neuroinflammatory phase of HTLV-1 infection in human (HAM/TSP), although we were enable to demonstrate the it role in the infection (159).

As above mentioned, AA metabolism *via* cyclooxygenase (COX)-1 and COX2 originate prostanoids, such as PGE_2_, PGD_2_, TXA_2_, PGI_2_ (160), some of them with opposite functions (161). After binding to the receptors EP1, EP2, EP3, EP4, PGE_2_ induces inflammation, apoptosis, and cell proliferation (140, 144, 145, 162). In this context, the role of PGE_2_ in viral infection has been documented (163). In influenza A virus infection, it was described that the augment of PGE_2_ secretion inhibits type I interferon release, macrophage apoptosis, antigen presentation and T cell mediated immunity, with consequent increase in virus burden (164). Using genetic and pharmacological tools to inhibit PGE_2_ production, it was shown an increase in the survival of lethally virus-infected mice, and PGE_2_ inhibition was suggested as a treatment for influenza A infection (164). More recently, in H1N1 infected obese mice, an immune suppressive role of PGE_2_ was also reported, and the investigators suggested the treatment with paracetamol to restore cytokine production and to rescue lethally H1N1 infected mice from death (165).

Considering the participation of lipid mediators in COVID-19, eicosanoids and docosanoids could be markers of disease severity (166, 167). Overmyer and coworkers quantified and compared metabolites and lipids from COVID-19 and non-COVID-19 patients blood samples and reported that dysregulation of lipid transport and neutrophil degranulation were associated with severe COVID-19 (168). The diminished production of immune regulatory PGs and the increase of AA-derived products, 5-lipoxygenase-derived products (5-LOX) and cytochrome P450 (CYP450) species in the serum were associated with severe disease (166). By contrast, it was suggested that AA and others lipid mediators potentially could inactivate SARS-CoV-2 (169). A lipid storm within the lungs of severe COVID-19 patients was characterized by the oxylipids quantification on BALF of patients, under mechanical ventilation support. The authors showed a predominance of COX metabolites, such as TXB_2_, PGE_2_, 12-HHTrE and PGD_2_; LTs, notably LTB_4_, 20-COOH-LTB_4_, LTE_4_, and eoxin E4; 15-lipoxygenase metabolites derived from linoleic, arachidonic, eicosapentaenoic, and docosahexaenoic; and specialized pro-resolving mediators, such as lipoxin A_4_ and the D-series resolvins (170). These studies show that the role of lipid mediators in the SARS-CoV-2 infection requires further investigations.

## HLA-G and COVID-19

The genetic susceptibility to intracellular pathogens has been demonstrated for a variety of infectious diseases, such as leprosy, tuberculosis, malaria and noteworthy those caused by RNA viruses, such as HIV-1/AIDS, hepatitis B and C and dengue (171), including SARS-CoV (172, 173). In that sense, it has been reported that the host genetic polymorphisms such as ABO, Human Leukocyte Antigen (HLA), IL-6, complement and angiotensin-converting enzyme 2 (ACE-2) genes may be associated with SARS-CoV-2 susceptibility and COVID-19 severity (15, 174–178). Understanding the contribution of the host genomics as an intrinsic factor to the variable outcomes of SARS-CoV-2 infections, from asymptomatic to severe form or death, will enable to improve the medical practice and patient care. For this, it is necessary to translate the host genetic variations into predictive risk factors that will foster the clinical management.

Human leukocyte antigens (HLA) are involved in several important functions of the immune system, starting from antigen presentation to lymphocytes, performed by the classical class I (HLA-A/B/C) and class II (HLA-DR/DQ/DP) histocompatibility molecules), and extending until the control of the immune response, primarily performed by the non-classical class I molecules (HLA-E/F/G) (179). Among these molecules, HLA-G is the most studied, and its major function is the down-regulation of the function of the innate and adaptive immune system cells, by means of the interaction with inhibitory receptors (180). Accordingly, HLA-G may inhibit the proliferation of T and B lymphocytes, the activity of antigen-presenting cells (APC) and the cytotoxicity of CD8^+^ T cells and NK cells (181, 182). Because of these properties, HLA-G has been recognized as a major immune check point molecule. In certain conditions, like viral infections, the overexpression of HLA-G can create a tolerogenic environment; inhibiting several steps of the immune response, propitiating the spreading of infectious (183).

HLA-G is encoded within the human Major Histocompatibility Complex (NCBI gene ID: 3135), and in contrast to normal conditions in which the expression of HLA-G is very restricted to few tissues, ectopic HLA-G expression may occur in pathological conditions, such as viral infection, favoring the virus escape from the patient’s immune surveillance (184). Several HLA-G isoforms have been described that can be expressed on cell membranes and reach distant immunological sites as soluble forms (sHLA-G). Both membrane-bound and sHLA-G induce regulatory mechanisms, such as apoptosis of CD8^+^ T and NK cells, inhibition of B-cell proliferation, differentiation and antibody secretion (185).

Overall, all segments of HLA-G may contribute to its immunoregulatory properties. The short cytoplasmic tail retains HLA-G longer in the endoplasmic reticulum and prolongs the half-life of the molecule on the cell surface, because of the lack of an endocytosis motif, permitting multiple interactions with cells of the immune system. The extracellular domains interact with leukocyte receptors, including CD8, ILT-2/LILRB1, and ILT-4/LILRB2, and the NK immunoglobulin-like receptor KIR2DL4 (CD158d) (183).

Although HLA-G may interact with KIR molecules, the most important interaction are with the LILRB1s (expressed on the surface of NK and lymphomononuclear cells) and LILRB2s (expressed on the surface of monocytes and dendritic cells) (186). Both LILRB1 and LILRB2 have several ITIM receptors in their cytoplasmic tail, inhibiting signaling events triggered by stimulatory receptors (187). Both receptors interact with classical HLA class I molecules; however, the binding with HLA-G presents three- to four-fold higher affinity when compared to classical molecules (188). The CD8 molecule also interacts with the α3 domain of classical and non-classical molecules, including HLA-G and HLA-E, although exhibiting different affinities (183).

Besides the inhibitory role of HLA-G in cytotoxic cells exhibiting CD8 on their surface, other interactions of HLA-G molecules with CD8 cells deserve attention. Soluble HLA-A-B-C and -G molecules can induce apoptosis in activated CD8^+^ T lymphocytes as well as in CD8^+^ NK cells (lacking the T cell receptor) at similar rates. The binding of soluble HLA molecules to CD8 leads to apoptosis, upregulating Fas production and Fas/FasL interaction (189, 190). Classical and non-classical soluble molecules may produce similar effects on apoptosis of CD8^+^ cells; however, in conditions in which soluble HLA-G is elevated, including pregnancy, some tumors and allografts with good prognosis, this mechanism may represent an additional immunomodulatory effect of HLA-G. Although the precise mechanism of this interaction has not been elucidated, the interaction of soluble forms of HLA-G containing the α3 domain is plausible (183). Taken together, these observations suggest that, in the context of viral infections, the expression of HLA-G is a complex process modulated by many factors such as HLA-G polymorphism, stage of infection, drug therapy, and cytokine expression patterns, which may contribute to an immunological environment affecting the outcome of infection (191).

Viruses have also evolved several strategies to evade the cytotoxic effect of immune effector cells, including downregulation of HLA classical class I molecules and the upregulation of non-classical molecules, or both. As a result, the increased HLA-G expression, induced by the virus itself or by the presence of an inflammatory milieu containing transcription and post-transcription factors that positively modulate HLA-G expression, may exacerbate virus morbidity and/or patient mortality, the expression of HLA-G in these disorders may predict a worse outcome and greater susceptibility to cell transformation (192). Specifically in the SARS-CoV-2 infection, few studies have been conducted so far. Zhang and coworkers showed that HLA‐G expression in peripheral T cells, B cells and monocytes reflected a high, low, high pattern, respectively, to viral replication and clearance, suggesting that SARS‐CoV‐2 infection might be involved in the regulation of HLA‐G expression, which impairs CD8^+^ cell‐mediated recognition and supports a mechanism of immune evasion (193). Zidi suggested that the decrease of HLA-G expression on cell surface could be a consequence of cleavage by MMP. The author still suggested that the follow-up of soluble HLA-G could be used to identify a worse prognostic of COVID-19 among patients with high levels of soluble HLA-G, and therefore the blocking of HLA-G interaction with their receptors may enhance immune response (194).

Although many clinical trials have been conducted, presently, no effective drug treatment is available for SARS‐CoV‐2. However, host humoral and cellular immune responses are imperative to control viral infections. In this review article, we discuss the role important soluble mediators, antibodies, and leukocytes that are involved on the pathogenesis of SARS‐CoV‐2-mediated infection and on the outcome of COVID-19 patients. In this scenario, we highlight the role the well-recognized tolerogenic role of the HLA‐G molecule.

## Concluding Remarks

From the identification of SARS-CoV-2 in January and February to COVID-19 being declared a pandemic by WHO in March 2020, we have followed the movement of the Chinese scientific community in the dissemination of clinical and laboratory findings of mild and severe clinical forms of this new disease that mobilized the world in a way that we had not yet witnessed. As the SARS-CoV-2 infection spread to different continents and countries, we have followed the rapid mobilization of the other scientific communities worldwide, which seek a better understanding of the generation of the immune response, leukocyte populations and soluble mediators involved in the protective response or unfavorable disease outcome. From April 2020, we observed an increasing number of studies focused on the description of pulmonary immunopathology and the functional characterization of circulating leukocyte populations. Undoubtedly, a huge advance in the characterization of infected cells, with studies that were initiated with cell lines and advanced to the autopsies of lung tissue of deceased individuals, suggesting that ACE-2 receptor may not be the only cell entry for the virus. From now on, it will be essential to investigate how immunopathology is generated and the mechanisms behind the damage tolerance dysregulation. In this sense, the study of the main comorbidities that participate of COVID-19 immunopathology will be essential to understand the different clinical outcomes. For this, experimental models and well-defined experimental approaches *in vitro* will have an important role for the understanding of pulmonary immunopathology to advance from the descriptive phenomenon to the investigation of the triggering mechanism. In this scenario, researchers should be able to identify targets for therapies aimed at reducing pulmonary immunopathology and biomarkers that make it possible to differentiate mild, moderate, severe, and critical clinical forms, and that make it feasible to monitor the evolution of the disease.

## Author Contributions

VB participated in the conceptual design. TF-S, MD-B, and VB drafted, wrote, reviewed, and edited the manuscript. SM reviewed and edited transcriptome section. ER reviewed and edited the manuscript. AF reviewed and edited HLA-G section. CC reviewed and edited sex steroid hormone section. CS and LF reviewed and edited lipid mediator section. All authors contributed to the article and approved the submitted version.

## Funding

The authors gratefully acknowledge São Paulo Research Foundation - FAPESP (2020/05270-0, 2020/05207-6, and 2017/21629-5) and National Council for Scientific and Technological Development - CNPq (312606/2019-2, 311639/2017-8, and 309583/2019-5).

## Conflict of Interest

The authors declare that the research was conducted in the absence of any commercial or financial relationships that could be construed as a potential conflict of interest.
